# Validation of a Multiclass Method for the Screening of 15 Antibiotic Residues in Milk Using Biochip Multi-array Technology and Its application to Monitor Real Samples

**DOI:** 10.22037/ijpr.2021.114359.14813

**Published:** 2021

**Authors:** Hassan Yazdanpanah, Arash Mahboubi, Samira Eslamizad, Zakieh Karimi, Elham Rashidi, Jamshid Salamzadeh

**Affiliations:** a *Food Safety Research Center, Shahid Beheshti University of Medical Sciences, Tehran, Iran. *; b *Department of Toxicology and Pharmacology, School of Pharmacy, Shahid Beheshti University of Medical Sciences, Tehran, Iran.*

**Keywords:** Sulphonamides, Validation, Screening, Milk, Decision 2002/657/EC, Biochip, Multi-array

## Abstract

Veterinary drugs are extensively and legally consumed to treat and prevent disease in chattels but some are also used illegally as growth-stimulating agents. Inappropriate or intensive use of antibiotics can cause allergic reactions and, above all, antibiotic resistance. A multiclass approach for the screening of antimicrobial substances in milk was validated in consonance with Commission Decision 2002/657/EC and to the European guideline for the validation of screening methods for veterinary medicines. This biochip-based approach enables the simultaneous determination of a total of 13 sulphonamide, dapsone and trimethoprim. For monitoring of antibiotic residues, 53 UHT milk samples collected from Tehran, IR Iran were screened applying this technology. The result showed that for all antibiotic residues, the positivity threshold T was much more than the cut-off value Fm. A false positive rate of less than 5% was found for all antibiotics which are satisfactory. All detection capabilities (CC*β*) were well below the Maximum Residue Level (MRL) set by the European Commission (100 *µ*g/kg for the sum of all sulphonamides and 50 *µ*g/kg for trimethoprim in milk). The screening results of 53 milk samples showed that 71.7% of samples were compliant and all positive samples were below the MRL set by European Commission. This study showed that the biochip-based technique is valid to identify and quantify antibiotic residues in milk at the studied validation levels. The method was rapid, easy, safe, and able to screen 13 sulphonamide, dapsone and trimethoprim from a single milk sample simultaneously with no sample preparation procedure (or just one-step centrifugation).

## Introduction

Antimicrobial drugs have been used in dairy products for more than five decades in dairy cattle production to prevent and treat livestock diseases, for instance, mastitis, pulmonary diseases and diarrhea or to increase milk production (1–4). However, the incorrect practices of antimicrobial drugs result in antibiotic residues in milk, that cannot be completely destroyed with heat treatment and are easily transported from the raw milk into milk products (3). Allergic reactions can cause in sensitive persons due to the accumulation of veterinary antibiotics in edible tissues. Additionally, using low levels of veterinary antibiotics in the long term could increase antibiotic-resistant bacteria (1, 5). 

To make sure these antibiotic residues do not pose any health risk to users, the European Union (EU), U.S. Food and Drug Administration, and other international regulatory authorities established MRLs for several antibiotics in foodstuffs of animal sources, including milk (1, 6–8). EU has set safe MRLs based on the sum as the parent compound of all present sulphonamides. For milk, the maximum allowable total residue concentration is set at 100 μg/kg for the sum of all the sulphonamides. For trimethoprim (TMPM), an MRL of 50 μg/kg in milk has been established. Dapsone (DAP) is a prohibited substance in all matrices (6). Considering these MRLs, to detect low levels of these compounds in milk samples, selective and sensitive analytical methods are highly suggested (8). Varied techniques have been applied for the detection of antibiotic residues in milk, which are classified mostly as chromatographic (51.34%), immunological (25.89%), microbi-ological (16.96%), and miscellaneous (8.04%) (9). The chromatographic technique is inc-reasingly being used, due to the higher rate, higher sensitivity, specificity, and quantification capability. On the other hand, various immunological and microbiological techniques can be used at a cheaper rate and rapidly with lesser efficiency (9). The Evidence Investigator™ Biochip Array Technology (Randox, UK) is used for performing quantitative detection of multiple analytes from a single sample simultaneously. The core biochip technology is a solid substrate containing an array of distinct test regions of immobilized specific antibodies for different antibiotics. A competitive chemiluminescent immunoassay is employed (10).

This study describes the validation of an innovative system, Evidence Investigator found on biochip array technology, for the detection of 13 sulphonamide (Sulphadimethoxine (SDIM), Sulphadiazine (SDZ), Sulphadoxine (SDX), Sulphachlorpyridazine (SCPD), Sulphamethoxypyridazine (SMPD), Sulphisoxazole (SSX), Sulphathiazole (STZ), Sulphaquinoxaline (SQX), Sulphapyridine (SPD), Sulphamerazine (SMZ), Sulphamethoxazole (SMXZ), Sulphamonomethoxine (SMMX), Sulphamethazine (SMTZ), DAP and TMPM residues from a single milk sample and its application on real Ultra-High-Temperature (UHT) milk samples, in consonance with Commission Decision 657 published in 2002 and the European guideline about validation of screening methods for residues of veterinary medicines (11, 12). Up to now, to our knowledge, no article was published about the validation of 13 sulphonamide and DAP and TMPM residues in milk with an Evidence Investigator™ system.

## Experimental

*Chemicals and reagents*

SDIM, SDZ, SDX, SCPD, SMPD, SMZ, SSX, STZ, SMTZ, SQX, SPD, SMXZ, SMMX, DAP, and TMPM were obtained from Sigma-Aldrich (Switzerland) and Supelco (USA). Antimicrobial *I* Ultra Array (AM *I* Ultra) kit (Cat.No.EV 3843) and milk preparation Kit (Cat.No. EV 3776) were purchased from Randox Food Diagnostics (UK).

*Apparatus*

Centrifuge Rotinta 380R (Hettich, Germany), Vortex model Hei-MIX Reax top (Heidolph, Germany) and roller mixer model BMW-4-1-10-R-1-89 (Behdad, IRAN), Evidence Investigator biochip analyzer (Randox Food Diagnostics, UK).

*Blank and real milk samples*

Twenty long life and skimmed milk, bio-milk (fresh containing 3.5% fat), and bio-milk (long life containing 3.5% fat) samples were obtained from the UK and Austria as blank samples. Fifty-three UHT treated and homogenized milk samples with different content of fat (15 low fat, 17 semi-fat and 21 full-fat) were collected from retail stores and supermarkets in Tehran during July and August 2017. Full fat milk samples contain 3% fat, semi fat or semi-skimmed milk samples contain 2.5% fat and skimmed or low-fat milk samples contain 1.5% fat. These samples were produced in some cities of Iran such as Tehran, Karaj, Amol, Ghazvin, Shahrekord and Arak. After collection, the samples were stored at 2-8 ºC until analysis.

*Standard solutions preparation*

The concentration of each antibiotic stock solution except SQX was 1 mg/mL in methanol and the concentration of SQX was 0.5 mg/mL in a mixture of methanol and DMSO (1:1). For each antibiotic, intermediate standard solutions with concentrations of 10 *µ*g/mL in methanol were prepared. For preparing working solutions of each compound, the intermediate standard solution was further diluted. 

*Sample preparation*

For skimmed milk, no sample preparation was required. Full fat and semi-skimmed milk samples were centrifuged before spiking and applying to the biochip. For spiking at different concentrations, 100 *µ*L of the working solution was added to 900 *µ*L of the blank milk. The full fat and semi-skimmed samples were centrifuged for 10 min at 2880 rcf (skimmed milk samples do not require centrifugation). The lower layer (under the fat layer) of samples was diluted with working strength wash buffer (1:1). 

*Evidence Investigator system*

*Multi-array biochip technology*

Antimicrobial *I* Ultra Array kit applied to the Evidence Investigator was used for the simultaneous biochip-based immunoassays (Randox Food Diagnostics, UK.). 

Biochip Array Technology provides a chemically activated 9x9 mm ceramic biochip as a solid-phase reaction vessel. Biochips are pre-fabricated with discrete test regions (DTR’s); a different antibody is immobilized at each spatially distinct DTR. For simultaneous detection of sulphonamides (SDIM, SDZ, SDX, SCPD, SMPD, SMZ, SSX, STZ, SMTZ, SQX, SPD, SMXZ, SMMX), DAP and TMPM a competitive format is employed. Horseradish peroxidase (HRP)-labeled conju-gate is applied; when this is captured by the related antibody, a complex is formed that outputs light upon the addition of a signal reagent. Any target analyte present in the samples will compete with enzyme-labeled conjugate for complexation and enhanced levels of antimicrobials in a specimen will cause reduced binding of conjugate and thus the chemiluminescence signal emitted will decrease. The light signal produced from each of the test regions on the biochip is detected using digital imaging technology and compared to that from a stored calibration curve. The concentration of each existing analyte in the sample is calculated from the calibration curve (10, 13).

Individual biochip carriers contained nine biochips, which are also the vessels where the immunoreactions take place for individual samples. The immunoassays were performed following the manufacturer’s instructions. Briefly, 200 *µ*L of assay diluent followed by 50 *µ*L of calibrator/sample were pipetted per biochip. For mixing reagents, all edges of the handling tray (with the capacity to accommodate 6 carriers) were taped gently. The handling tray was incubated for 30 min at +25 °C and 370 rpm. Then 50 *µ*L of working strength conjugate was added to each biochip and incubated for 60 min at +25 °C and 370 rpm. Afterward, wash cycles were carried out and after the final wash, any residual wash buffer was removed. Working signal reagent (250 *µ*L) was then added to each and the biochip covered to protect from light. After exactly 2 min (±10 s) the biochip carrier was placed into Evidence Investigator and images were captured by the software.

*Image and data processing*

The biochip detection is based on a chemiluminescent signal with a CCD (charge-coupled device) camera, which records the light emission simultaneously from whole the separate test sites on every biochip on each biochip carrier. The system incorporates dedicated software to process and archive the multiple data generated.

*Validation procedure*

The validation was carried out in accordance with the European guideline regarding the validation of screening methods that is based on the principles of European Decision No 2002/657/EC (11, 12). The performance criteria including CC*β*, practicability, applicability, specificity and stability were evaluated.

*Number of samples required for validation*

As stated by the European guideline (11,12) if the screening target concentration is considered at half the Regulatory Limit or lower (*e.g.* 1/2 MRL), 20 “Screen Positive” Control Samples (with one or no false compliant result) is adequate to prove that CC*β* is less than the MRL.

*Identification of the Cut-Off Level and calculation of CCβ*

The MLRs, calibration ranges, and spike levels are indicated in Table 1. Validation of screening methods (whether qualitative or semi-quantitative) necessitates identification of a cut-off level, which indicates that a sample contains an analyte at or above the screening target concentration

(12). The cut-off level and CC*β* were determined for the 13 sulphonamide, DAP and TMPM. Our data were calculated on the signal in RLU. 

For each antibiotic residue tested the average value and the SD of the signal (for the 20 blank samples and the 20 spiked samples) were calculated. 

The threshold value T was calculated from the blank samples as follows:

T = mean RLU signal of the blank samples - 1:6 × SD RLU signal of the blank samples

The cut-off factor Fm was calculated from the samples spiked with 15 antibiotic residues as follows:

Fm = mean RLU signal of the spiked samples + 1:64 × SD RLU signal of the spiked samples

If the cut-off value Fm is below the T, the target concentration during the validation is identified for the determination of detection capabilities (CC*β*). Otherwise, if the cut-off value Fm is not below the threshold T, the concentration of antibiotic residues in the validation step should be increased. 

*Practicability*

The purpose of practicability is to survey whether the procedure is suitable or not for repetitive analysis. Relevant aspects in this respect are the time needed for each analysis, the skills of the user of the method, essential equipment (usual or specific equipment in a lab), instruments (particular or usual instruments in a lab), reagents (ready to use or not) and environmental conditions (wide or narrow temperature intervals to use the kit) and the number of antibiotics of interest.

*Applicability*

Milk samples were selected to provide an indicative group of varying degrees of fatness, duration of storage, and different places of production. The applicability of the kit was evaluated by determining CC*β* of the 13 sulphonamide, DAP, and TMPM for 20 different spiked samples in varied types of milk (skimmed, semi-skimmed and full-fat and fresh or long-life). 

*Stability in the matrix and pure solvent*

The stability of different analytes in the matrix and the pure solvent was obtained through a literature review.

*Application of this method on real samples*

To establish the capability and suitability of the validated method, the method was applied to 53 UHT treated and homogenized milk samples for simultaneous determination of 13 sulphonamide, DAP and TMPM.

## Results

*Detection capabilities*

**Figure 1:**
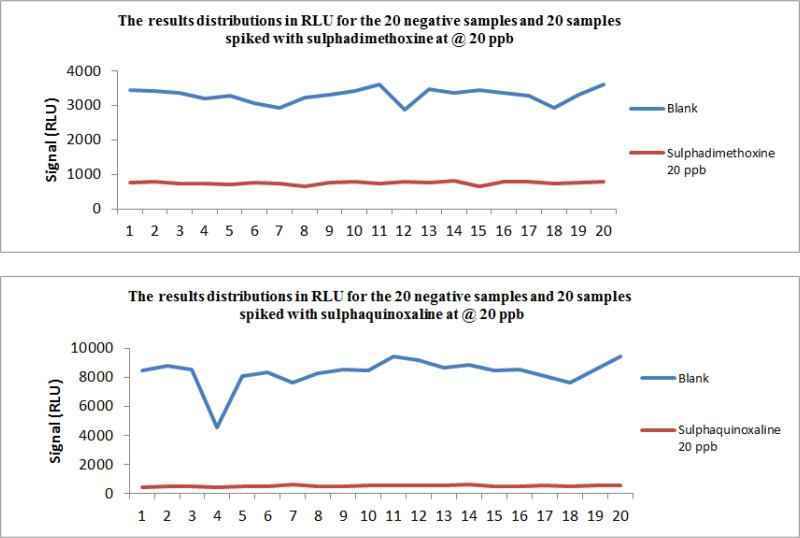
The result (in RLU of the 20 negative samples and the 20 spiked samples) for sulphadimethoxine and Sulphaquinoxaline

All of the compounds were detected on the first day of validation so the concentration of spike levels was not changed. The result (in RLU of the 20 negative samples and the 20 spiked samples with the fifteen antibiotic residues) for sulphadimethoxine and sulphaquinoxaline are presented in Figure 1. The result of the findings when Fm is considered as the cut-off value is shown in Table 2, the RLU values of T were much higher than Fm for all the compounds. 

An acceptable rate of false-negative results of 5% was acquired for 13 sulphonamide, DAP and TMPM, indicating that the result is compliant, so according to Commission Decision 2002/657/EC, the validated concentration is equal to CC*β* (Table 3) (11, 12). 

*Practicability*

No sample preparation was required for skimmed milk. Semi-skimmed and full-fat milk samples only required one step centrifugation before applying to the biochip. A small sample volume was required (50 *µ*L). The amount of material provided in the kit was enough with an easy to use procedure. The software was simple in its functionality. The results were available in ppb and RLU. The data disk was present in the box and when a fresh batch number was used, it was inserted and loaded.

*Specificity and false-positive rate*

During validation, the 20 blank and 20 spiked milk samples were analyzed after 3 days. The results are presented in Table 4. If T was selected as the cut-off value, one sample out of 20 (5%) was screened false positive as indicated in Table 4 for SQX, SMTZ, SSX, SPD, SCPD and DAP, two samples out of 20 (10%) screened positive for SMXZ, SMZ, SMMX, SMPD, SDX and TMPM and three samples out of 20 (15%) for SDIM, no false-negative results were observed. If Fm was selected as the threshold value, one sample out of 20 (5%) was false negative for SDZ, SQX, SMXZ, SMZ, SMMX, SCPD, DAP and TMPM and no false positive results occurred. Fewer false negative results of the test when taking T as the cut-off value will be more sensitive, but the increase of the false positive rate will impose additional costs for confirmatory analyses of the compliant sample. With the selection of Fm as the cut-off level an agreement between detection capabilities, low enough to reach the relevant RC and a rational false positive rate was occurred. So, for deciding on the positivity of a sample, Fm was selected as the cut-off value (Table 2). 

*Applicability*

During the validation procedure, the kit applicability for different types of milk (skimmed, semi-skimmed and full-fat and fresh or long-life) has been evaluated. The fat content and storage duration of milk did not affect the result. The AM *I* Ultra Array kit was then applicable to a wide range of milk samples.

*Stability of antibiotic residues*

The stability of antibiotic residues was presented in some studies. Chen *et al. *investigated the stability of 8 sulphonamides, including sulphapyridine, sulphaguanidine, sulphamonomethoxine, sulphamethoxazole, sulphadiazine, sulphachlorpyridazine, sulphadimethazine, and trimethoprim, in raw milk under different conditions. Their result showed that most sulphonamides were stable entirely (recovery = 90% ~ 120%) in -80 °C, -20 °C, 4 °C within 30 d, 30 d, 48 h respectively (14).

Other literature investigated the degradation of eight sulphonamides (SDZ, STZ, SPD, SMZ, SMTZ, SCPD, SDIM and SQX) in skimmed milk, after application of kinetic equations for different heat conditions used in dairy processing showed that sulphonamides are very stable during pasteurization (63 °C for 30 min and 72 °C for 15 s) as well as UHT sterilization (140 °C for 4 s) (15). Laszlo *et al*., studied stability of different antimicrobial drugs in heat, their research showed that sulphonamides (sulphathiazole, sulphadiazine) are more heat-stable antibiotics and acted rather similarly to each other showing high to intermediate stage of heat stability (16). Traub and Leonhard showed that trimethoprim revealed to be heat-stable in aqueous solution and at higher concentrations when autoclaved at 121 °C for 15 min (17).

*Analyses of real samples*

During the validation process and routine analyses, positive and negative quality controls (QCs) were used. During validation and analyses of the 53 real milk samples, spiked samples and control in the kit were used as positive QCs and blank samples used as negative QCs. When the QCs during analyses of real samples in each run were not valid, the samples were reanalyzed.

The results of the screening of the real milk samples are presented in Table 5. Samples with RLUs higher than the cut-off level were considered as presumptive negatives. Samples with RLUs lower than the cut-off level were considered as screening positive. The results indicate that only one sample was no compliant for SMTZ. 

In this study, the sum of sulphonamides and TMPM in all samples was below the MRLs set by the European Commission (100 *µ*g/kg, 50 *µ*g/kg respectively) (6).

## Discussion

Iran is a country with a long dairy tradition and has self-sufficiency in the milk of about 100% and this is why the government and the dairy sector are pointing at exporting milk to other countries (18). Dairy production in Iran has increased to a level of about 9,000,000 tons of milk per year (18).

Although antimicrobial drugs are beneficial for the treatment of infections, their occurrence in milk reasons adverse public health effects such as drug resistance and hypersensitivity that could be life-threatening (4).

Various screening and confirmatory methods are existing for the detection or the determination of antibiotic residues in milk. Confirmatory methods are chromatography methods, high-pressure liquid chromatography (HPLC) and mass spectrometry (MS). Screening methods are used as a first choice to detect the occurrence of antibiotic residues in food of animal origin (19), immune assays and microbiological are widely used because of their low cost and short time of analysis (9). Comparison of various commercial kits or the screening methods of antimicrobial drug residues in milk is presented in Table 6. The AM*I* Ultra Array kit evaluated reports specific results for each sulphonamide similar to chromatographic methods.

This study has shown that AM *I* Ultra kit is valid as a screening method to detect and identify antibiotic residues in milk at the studied validation levels. All CC*β* values were well below the MRLs set by the European Commission. The screening results of 53 authentic milk samples showed that 71.7% of samples were compliant. The method was created to be easy, rapid, safe and able to screen simultaneously 15 antibiotic residues from a single milk sample of different types of milk with no sample preparation procedure (or just one-step centrifugation). 

Many studies have been conducted worldwide regarding antibiotic residue in milk samples. In a survey conducted by Bilandzic in Croatia (25), a total of 1259 raw milk samples were examined over three years for several antibiotics, their results showed that 0.69% of the total samples were positive. In another study, Bilandzic *et al. *reported that among 119 raw milk samples, none of them showed the presence of veterinary drug residues exceeding the maximum residues levels (MRLs) established by European Union and Croatian legislation (24). In a survey carried out in Romania, out of 2785 total milk samples, 124 (4.45%) were found to be contaminated with antibiotic residues,130 samples were uncertain (±) (4.67%) and 2531 samples (90.88%) were free of antibiotic residues (26). Nikolic *et al. *(23) tested during six months 6161 samples of raw milk, collected from Montenegro dairies and it was found that 478 samples or 7.84 % were positive. In Slovenia, a total of 3358 milk samples were analysed and most of them (99.4%) were negative (24). By contrast, sulphonamides (18.4%), tetracyclines (48.9%), and quinolones (6.8%) were found in milk samples from Macedonia, although drug residues were below the MRLs (27). Mottaghianpour *et al.* (28) analyzed 60 milk samples including industrial samples of different brands and local raw milk samples were collected from the Zanjan market, about 31% and 9% samples of industrial and local raw milk samples had antibiotic residue above MRL. In heated (pasteurized and sterilized) milk samples were sulphonamides (about 31%) and in local raw milk, samples were beta-lactam (about 8%) and tetracycline (1%) antibiotics respectively. Rahimi *et al.* (29) concluded that out of 80 total cow milk samples, 12% contaminated by sulphonamides and the mean concentration of sulphonamides residues in the samples was 41.44 ng/g. In a study of 100 raw cow milk samples in Iran, Mollaei A *et al.* (30) reported that 95% of samples (95) were antibiotic-free and 5% (5) contained antibiotic residual. 

All these studies indicate the importance of controlling and monitoring milk production worldwide. The present study showed screening results <MRLs in milk samples. Comprehensive studies are needed to monitor antibiotic residue in milk produced in different provinces in IR Iran.

**Table 1:** Maximum Residue Limit (MRL), calibration range and spiking levels of 13 sulphonamide, DAP and TMPM

**Compounds**	**MRL (EU)** ^*^ **(ppb)**	**Calibration range (ppb) regarding dilution factor (2)**	**Chosen spike level (ppb)**
Sulphadiazine (SDZ)	100	0-40	20
Sulphadimethoxine (SDIM)	100	0-40	20
Sulphaquinoxaline (SQX)	100	0-40	20
Sulphamethazine (SMTZ)	100	0-40	20
Sulphamethoxazole (SMXZ)	100	0-25	12
Sulphathiazole (STZ)	100	0-40	20
Sulphisoxazole (SSX)	100	0-40	20
Sulphapyridine (SPD)	100	0-40	20
Sulphamerazine (SMZ)	100	0-40	20
Sulphamonomethoxine (SMMX)	100	0-240	50
Sulphamethoxypyridazine (SMPD)	100	0-40	20
Sulphachlorpyridazine (SCPD)	100	0-40	20
Dapsone (DAP)	PS^**^	0-40	20
Sulphadoxine (SDX)	100	0-40	20
Trimethoprim (TMPM)	50	0-20	10

^ *^European Union. ^**^ Prohibited Substance.

**Table 2:** The summary results when Fm is considered as the cut-off value

	**SDZ**	**SDIM**	**SQX**	**SMTZ**	**SMXZ**	**STZ**	**SSX**	**SPD**	**SMZ**	**SMMX**	**SMPD**	**SCPD**	**DAP**	**SDX**	**TMPM**
Concentration (*µ*g/kg)	20	20	20	20	12	20	20	20	20	50	20	20	20	20	10
T value (RLU)	4282.65	2961.23	6664.50	2678.30	2722.14	6235.40	3186.33	2337.10	5598.27	1706.54	3049.47	6689.48	4207.31	2421.01	1292.80
Fmvalue (RLU)	406.78	825.57	614.27	380.34	291.84	268.31	280.70	419.48	438.78	572.06	473.66	1178.84	826.78	431.28	260.65
T > Fm	Yes	Yes	Yes	Yes	Yes	Yes	Yes	Yes	Yes	Yes	Yes	Yes	Yes	Yes	Yes
Number of FP	0	0	0	0	0	0	0	0	0	0	0	0	0	0	0
FP rate (%)	0	0	0	0	0	0	0	0	0	0	0	0	0	0	0
Number of FN	1	0	1	0	1	0	0	0	1	1	0	1	1	0	1
FN rate (%)	5	0	5	0	5	0	0	0	5	5	0	5	5	0	5

RLU: Relative light Unit; SDZ: sulphadiazine; SDIM: sulphadimethoxine; SQX: sulphaquinoxaline; SMTZ: sulphamethazine; SMXZ: sulphamethoxazole; SCPD: sulphachlorpyridazine; SSX: sulphisoxazole; SPD: sulphapyridine; SMZ: sulphamerazine; SMMX: sulphamonomethoxine; SMPD: sulphamethoxypyridazine; STZ: sulphathiazole; DAP: Dapsone; SDX: sulphadoxine; TMPM: Trimethoprim; FN: false negative; FP: false positive.

**Table 3:** Detection capabilities CC*β*

	**SDZ**	**SDIM**	**SQX**	**SMTZ**	**SMXZ**	**STZ**	**SSX**	**SPD**	**SMZ**	**SMMX**	**SMPD**	**SCPD**	**DAP**	**SDX**	**TMPM**
LOD (*µ*g/kg) (as per manufacturer)	0.5	0.6	0.5	2.5	0.5	0.5	0.5	0.5	0.5	2	2	0.5	0.5	0.5	0.5
Spike level used for validation (*µ*g/kg)	20	20	20	20	12	20	20	20	20	50	20	20	20	20	10
CC*β* (*µ*g/kg)	20	20	20	20	12	20	20	20	20	50	20	20	20	20	10

**Table 4:** The number of false-negative and false-positive results depending on the selection of the cut-off factor Fm or threshold value T as cut-off level

		**SDZ**	**SDIM**	**SQX**	**SMTZ**	**SMXZ**	**STZ**	**SSX**	**SPD**	**SMZ**	**SMMX**	**SMPD**	**SCPD**	**DAP**	**SDX**	**TMPM**
T value (in RLU) (n = 20)		4282.65	2961.23	6664.50	2678.30	2722.14	6235.40	3186.33	2337.10	5598.27	1706.54	3049.47	6689.48	4207.31	2421.01	1292.80
Cut-off = T (n = 20)	False positive	0	3	1	1	2	0	1	1	2	2	2	1	1	2	2
	False negative	0	0	0	0	0	0	0	0	0	0	0	0	0	0	0
Fm value (in RLU) (n = 20)		406.78	825.57	614.27	380.34	291.84	268.31	280.70	419.48	438.78	572.06	473.66	1178.84	826.78	431.28	260.65
Cut-off = Fm (n = 20)	False positive	0	0	0	0	0	0	0	0	0	0	0	0	0	0	0
	False negative	1	0	1	0	1	0	0	0	1	1	0	1	1	0	1

RLU: Relative light Unit; SDZ: sulphadiazine; SDIM: sulphadimethoxine; SQX: sulphaquinoxaline; SMTZ: sulphamethazine; SMXZ: sulphamethoxazole; SCPD: sulphachlorpyridazine; SSX: sulphisoxazole; SPD: sulphapyridine; SMZ: sulphamerazine; SMMX: sulphamonomethoxine; SMPD: sulphamethoxypyridazine; STZ: sulphathiazole; DAP: Dapsone; SDX: sulphadoxine; TMPM: Trimethoprim; FN: false negative; FP: false positive.

**Table 5:** Occurrence of sulphonamides, DAP, and TMPM in UHT treated and homogenized milk samples in RLU

**Parameters**	**SDZ**	**SDIM**	**SQX**	**SMTZ**	**SMXZ**	**STZ**	**SSX**	**SPD**	**SMZ**	**SMMX**	**SMPD**	**SCPD**	**DAP**	**SDX**	**TMPM**
Number of samples	53	53	53	53	53	53	53	53	53	53	53	53	53	53	53
Cut-off	406.78	825.57	614.27	380.34	291.84	268.31	280.70	419.48	438.78	572.06	473.66	1178.84	826.78	431.28	260.65
Number of positive samples	0	0	0	1	0	0	0	0	0	0	0	0	0	0	0
%Positive samples (%)	0	0	0	1.88	0	0	0	0	0	0	0	0	0	0	0

SDZ: sulphadiazine; SDIM: sulphadimethoxine; SQX: sulphaquinoxaline; SMTZ: sulphamethazine; SMXZ: sulphamethoxazole; SCPD: sulphachlorpyridazine; SSX: sulphisoxazole; SPD: sulphapyridine; SMZ: sulphamerazine; SMMX: sulphamonomethoxine; SMPD: sulphamethoxypyridazine; STZ: sulphathiazole; DAP: Dapsone; SDX: sulphadoxine; TMPM: Trimethoprim.

**Table 6.** Comparison of various commercial kits or the screening methods of antimicrobial drug residues in milk

**Group of drugs detected**	**Number of tested antibiotic residues (Sulphonamides)**	**Commercial kit (manufacturer, country)**	**Principle of the test (Type of reaction)**	**LOD (μg/kg)**	**Time per analysis**	**Special equipment**	**Reference**
Sulphonamides, DAP and TMPM	15 (13)	AM*I* Ultra (Randox)- UK	Chemiluminescent immunoassays on biochip surface	SDIM (0.6), SMTZ (2.5), SMMX (2) and others (0.5)	Approx.2.5 h	Evidence Investigator system	As per manufacturer
Wide range of antibiotics and Sulphonamides (SDZ and SMTZ)	14 (2)	BR-Test AS Brilliant, DSM, The Netherlands	Bacterial growth inhibition	SDZ (25-100), SMTZ (5-200) and others (3-1000)	2 h and 45 min	None	(20)
Beta-lactams, tetracyclinesSulphonamides and chloramphenicol	11 (1)	Charm II- Charm Sciences, Inc- London	Microbial or immunochemical	Sulphonamides (50-200)	Approx. 2 h 45 min	Charm II 7600 system	(21)
Beta lactams, Sulphonamides, Tetracyclines and others.	43 (9)	Copan Milk Test (CMT)- Copan Italia Spa- Italy	CMT combines the principle of agar diffusion with the reduction of an indicator pigment by microbial inhibition	Sulphonamides (50-150), others (2->10000)	Fixed time of 3 h	The C-Scan automatic reading system both for Microplate and Single Test versions	(20)
Wide range of antibiotics and Sulphonamides	19 (2)	Delvotest ampoules or SP-NT plates	Bacterial growth inhibition	Sulphonamides (100-250)	90 to 120 min	Delvotest	(22)
Wide range of antibiotics and Sulphonamides	31 (4)	Delvotest SP- DSM- The Netherlands	Bacterial growth inhibition	Sulphonamides (50-150), others (1-10000)	Reading time; 3 h	Delvoscan software available	(20)
Wide range of antibiotics and Sulphonamides	28 (6)	ECLIPSE 100- ZEU-INMUNOTEC- Spain	Bacterial inhibition growth	Sulphonamides (50-150), others (4-5000)	3.15-3.30 h	None	(20)
Sulphonamides	5	EIA kit (type 5101SULMp)- EuroProxima- The Netherlands	Competitive enzyme immunoassay ELISA	1.8	1.5 h	Microplate reader	(23)
Sulphonamides	-	Sulphonamides EIA 5101SULM1p- EuroProxima- The Netherlands	Competitive enzyme immunoassay ELISA	<2.5 ng/mL	1.5 h	Micro titre plate reader	(20)
Antibiotics and Sulphonamides	26 (6)	Euroclone KALIDOS MP- Euroclone Spa- Italy	Bacterial growth inhibition	Sulphonamides (100-200), others (2-3000)	3 h	None	(20)
SDIM, SMZ Beta lactams Neomycin Streptomycin Tetracyclines Quinolones	14 (2)	Parallux- MEDEXX Co., Ltd- Korea	Competitive EIA method	SMTZ and SDIM (10), others (2.3-75)	4 min	Parallux processor	(20)
Beta-lactams	12	Penzym®100 S- Neogen Corporation- USA	Enzymatic, colorimetric assay	2-70	22 min	None	(20)
Beta-Lactam and Tetracycline families	18	ROSA 3 Minute MRL Test for Beta-Lactam and Tetracycline Drugs- Charm Sciences, Inc.	Lateral Flow- Rapid receptor assay utilizing ROSA technology. 3-line reaction.	2-100	3 min	ROSA reader	(20)
Sulphonamides	19	HPTLC	chromatography	10 to 20 ng/mL	Approx. 3 h	HPTLC	(24)
Wide range of antibiotics and Sulphonamides	16 (2)	SCREENING PLUS- ZEU-INMUNOTEC- Spain	Bacterial inhibition growth	SMXZ (50), ST (5000), others (4-5000)	3.15-3.30 h	None	(20)
Beta lactams, Tetracyclines, SMTZ, Gentamicin	20 (1)	SNAP Test Kits – IDEXX- IDEXX LABORATORIES- USA	Enzyme-linked receptor-binding assay	SMTZ (At or below 10), others (2-111)	10 min	None or SNAPshot® reader	(20)
Tetracycline, oxytetracycline, chlortetracycline, doxytetracycline	4	TECNA – SuperScreen TETRA- TecnaS.r.l.- Italy	Binding assay	50	90 min (sample preparation not included)	None -Qualitative and quantitative	(20)
Wide range of antibiotics	32 (8)	Valio T 101 test- Valio Ltd- Finland	Bacterial growth inhibition	Sulphonamides (200-1000), others (2-5000)	4 h 30 mn	None	(20)

SDIM: sulphadimethoxine; SMTZ: sulphamethazine; SMXZ: sulphamethoxazole; SDZ: sulphadiazine; SMMX: sulphamonomethoxine; STZ: sulphathiazole.

## Conclusion

As far as we know, this is the first paper about the validation of the AM *I* Ultra Array kit in milk according to European guidelines. Antibiotic residues contamination in milk in addition to adverse health effects can also cause important financial losses for producers and manufacturers of milk and milk products.

This screening method was sensitive, rapid and able to screen 15 antibiotic residues in different kinds of milk simultaneously with a very simple experimental procedure. 

Although in this preliminary study the sum of sulphonamides, DAP, and TMPM in any of the samples were not higher than the EU MRLs, monitoring of these antibiotic residues in different types of milk in different seasons and other foods is necessary due to health and economic implications.
